# Media for Dimensional Stabilization of Rubber Compounds during Additive Manufacturing and Vulcanization

**DOI:** 10.3390/ma14061337

**Published:** 2021-03-10

**Authors:** Welf-Guntram Drossel, Jörn Ihlemann, Ralf Landgraf, Erik Oelsch, Marek Schmidt

**Affiliations:** 1 Professorship Adaptronics and Lightweight Design in Production, Chemnitz University of Technology, Reichenhainer Straße 70, 09126 Chemnitz, Germany; adaptronik@mb.tu-chemnitz.de or; 2 Fraunhofer Institute for Machine Tools and Forming Technology IWU, Reichenhainer Straße 88, 09126 Chemnitz, Germany; 3 Chair of Solid Mechanics, Chemnitz University of Technology, Reichenhainer Straße 70, 09126 Chemnitz, Germany; joern.ihlemann@mb.tu-chemnitz.de (J.I.); ralf.landgraf@mb.tu-chemnitz.de (R.L.); erik.oelsch@mb.tu-chemnitz.de (E.O.)

**Keywords:** 3D rubber printing, additive manufacturing, support material, vulcanization tests

## Abstract

The current article proposes a concept for the additive manufacturing of rubber components using extrusion-based 3D printing, in which an additional medium is added to ensure the maintenance of shape within the elastomeric structure during the additive manufacturing process and in the subsequent vulcanization process. Specific requirements for the dimensional stabilization of the media were defined and suitable media were derived. Silicone rubber, molding sand, and plaster were examined in experimental vulcanization tests for their suitability as possible media with regard to shape retention. Selected rubber geometries made of NBR were embedded in these media to undergo the vulcanization process. The results show a significant influence of the media on the heating times. All media were able to ensure that the rubber geometries maintained their shape during vulcanization. Nevertheless, some side effects were found. The silicone rubber did not cure properly around the rubber sample. Therefore, it was difficult to remove it from the rubber after vulcanization. The molding sand caused an increased surface roughness on the rubber. Plaster changed the glossy surfaces at the beginning to a matte one after vulcanization and residuals were difficult to remove. However, all media can serve as stabilization media with specific changes.

## 1. Introduction

Additive manufacturing is a collective term for manufacturing processes in which a material is automatically applied layer by layer to a finished part or component. Compared to traditional production processes for polymers, like injection molding, additive manufacturing processes have far fewer geometrical design restrictions and offer an enormous flexibility with regard to the individuality of components [1]. Depending on the application and product, liquid, powder, or solid materials can be used [2,3]. A broad variety of processes have been developed for the different groups of polymer materials. The best-known examples are Fused Deposition Modeling (FDM), Stereolithography (SLA), Binder Jetting (BJ), or Selective Laser Sintering (SLS) [4,5,6]. Typical polymers are photosensitive liquids and thermoplastics, which can be mixed with other elements, including metal, wood, ceramic, or carbon fiber [3,7]. SLS is also applicable for numerous plastics in granular form. In addition, elastic polymers such as thermoplastic elastomers can be processed using the FDM process [4]. Other examples are liquid silicones, which can be processed by Liquid Additive Manufacturing (LAM) or Drop on Demand process, see, e.g., [8,9].

Depending on the application, it is important to weigh up the use of additive manufacturing or conventional manufacturing methods for the production of polymer components. Comprehensive reviews on this topic can, e.g., be found in Jiménez et al. [10] and Pereira et al. [11]. Accordingly, additive manufacturing has the advantages that designed 3D models can be quickly transformed into real components, that the geometric complexity of parts only plays a subordinate role, and also that design adaptations can be made promptly. Therefore, additive manufacturing processes are particularly suitable for prototypes, individual parts, small series, and highly customized parts [10,11]. Though, the anisotropy associated with the layer-by-layer manufacturing can lead to complex and previously unknown failure modes [12]. In contrast, conventional manufacturing entails higher costs at the start of a production, e.g., for the machine and the tools [10,11]. Moreover, the used tools limit the individuality of components. However, conventional manufacturing is beneficial for series production. Compared to additive manufacturing, significantly more parts can be produced in a shorter time, which also have a higher quality in terms of surfaces and tolerances. Furthermore, conventional manufacturing is technologically more advanced and can process more material classes than is currently possible with additive technologies [10,11]. This applies in particular to the material class of vulcanizable rubbers. Indeed, numerous rubber forms and components can be produced in series production using processes such as injection molding or compression molding [13,14,15].

In contrast to other polymer materials, the material group of vulcanizable rubbers cannot yet take advantage of the abovementioned benefits of additive manufacturing. The reason for that is the rheological material behavior of vulcanizable rubbers [16]. Actually, rubber material can be discharged from a nozzle for layer-by-layer build-up, e.g., via extrusion-based 3D printing [17,18]. However, built-up forms may collapse by their own weight at a critical layer height. In addition, simple 2D structures such as flat sheets would lose their shape during the vulcanization process at the latest. The increase in temperature of the material, necessary for the cross-linking of the macromolecules, causes a reduction in the viscosity of the rubber compound [16]. As a result, the built-up structure experiences a strong change in shape, which can lead to a complete loss of geometry.

In additive manufacturing of polymers, this problem is usually solved by supporting structures. For example, in the case of FDM processes overhangs are stabilized by supporting structures automatically generated via slicer software for 3D printing [19]. The material for the component and the support material can be the same or they can be different. In any case, the supporting material should be easily removable from the component after printing (e.g., by water-soluble polymers as support material). Current research on support structures deals with minimizing the amount of material needed or optimizing the support structures for easy removal [20,21]. Nevertheless, due to the different requirements resulting from the rheological behavior of rubber during printing and due to the subsequent vulcanization process, these approaches are not easily transferable to rubber compounds. Therefore, there are far more requirements for the supporting material.

In the literature, a unique approach for the additive manufacturing of rubber is described, in which a high-temperature thermoplastic is extruded parallel to carbon black-filled rubber compounds and aims to stabilize the form. The rubber compound is then vulcanized inside the thermoplastic mold in an autoclave [22,23]. The process is currently still under research and is carried out with high-temperature thermoplastics as described. The approach in the current article is similar in many ways. In contrast, this particular investigation uses media that can be processed at room temperature to avoid any temperature effects on the rubber compounds. These media have the function of ensuring the shape of the rubber components during additive manufacturing and in the vulcanization process.

For this purpose, the requirements for the media are initially defined in Section 2. Based on this, media are selected in Section 3, and the experimental investigations carried out are described. Section 4 contains the results of the investigations as well as assesses the suitability of the media.

## 2. Media Requirements for Dimensional Stabilization

In order to derive the requirements for the media, the possible additive manufacturing concept for rubbers must be considered. As described above, the general processing of rubber compounds via extruder technology is possible and forms the basis of the concept. Therefore, the challenge is not to develop a new technology, but rather to combine the existing knowledge of current technologies in a suitable concept. So-called media for dimensional stabilization (MDS) during additive manufacturing and vulcanization of rubbers can be available in different forms such as powder, granulate, filament, but also in liquid form. If the latter is the case, the liquid medium must have a high toughness for the supporting effect in the process or harden correspondingly fast. Furthermore, it can consist of one or more supplied components that are brought together before the nozzle and react (e.g., harden) after the nozzle outlet. The MDS can be processed using extrusion-based 3D printing, e.g., However, the process must be designed to be compatible with extrusion-based 3D printing of rubber compounds. Due to the existence of different rubber compounds and different vulcanization methods, there are different solutions for the additive manufacturing process and the used MDS. In principle, the MDS should completely enclose the rubber for the vulcanization process and thus form the molding tool for the rubber component.

**Figure 1:**
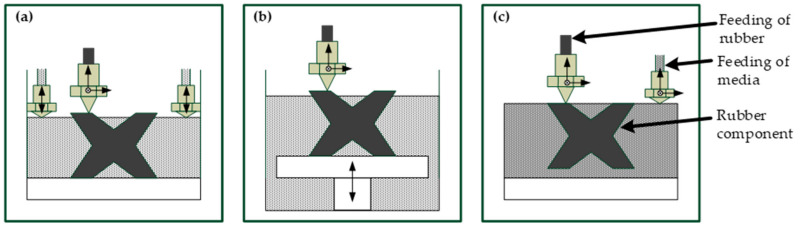
Possible variants of 3D printing process for rubber components with media for dimensional stabilization (MDS): (**a**) feeding a liquid medium via two vertically movable nozzles, (**b**) lowering of printed rubber layers into a liquid medium, and (**c**) feeding of a solid medium via a 3-axis movable nozzle.

Figure 1 shows three possible variants of how a 3D printing process with an all-enveloping medium for rubber compounds could look like. In Figure 1a, a liquid MDS is discharged through one or more nozzles. The rubber printing is a few layers ahead, so that the medium flows around and encloses the printed rubber layers. In this case, the medium should exhibit sufficiently good flow behavior and harden quickly for the stabilization effect. Figure 1b shows a variant with a moving printing bed. The liquid MDS is already completely in a reservoir. The rubber compound is printed above the media surface. After each finished printed layer, the printed component is lowered into the MDS by the height of one layer. In this case, the medium for stabilization should have a well-adjusted viscosity to fulfil the stabilization task while still being able to flow around the printed structure. In addition, the surface tension of the liquid should ensure a good wettability.

The solution in Figure 1c uses a solid medium. If supporting effects (e.g., overhangs) are necessary for the rubber component in the printing process, the medium should be built a few layers in advance. Otherwise, the layers for rubber and MDS should have almost the same height. Possibilities for the additive manufacturing of rubber shapes as described above are followed by the vulcanization process, which generates the final rubber component though cross-linking of the macromolecules. Different requirements for the MDS can be derived from the process steps “3D printing” and “vulcanization,” but also from the interaction of rubber and MDS.

### 2.1. Media Requirements during 3D Printing Process

The medium should completely enclose the printed rubber component. It should be liquid, almost liquid, or fine-pored during processing to ensure that it is completely adherent and produces smooth surfaces to the rubber shape. There shall be no air pockets in the medium. For the supporting effect of rubber structures, a sufficiently high viscosity or a fast hardening process after nozzle discharge is required. The processing temperature of the MDS is not permitted to reach the range of temperature in which the vulcanization could start.

### 2.2. Media Requirements during Vulcanization Process

For the vulcanization process, it is necessary that the MDS is temperature stable. The vulcanization temperature strongly depends on the material composition, in means of rubber and additives as well as fillers and reinforcing materials. However, temperatures of 200 °C are usually not exceeded [24]. For the medium, no significant changes in shape or volume fluctuations may occur. The expansion of enclosed air and the evaporation of water as well as other volatile components can cause bubbles in the rubber, and thus, porosity can occur. The medium must be able to guarantee a correspondingly high counterpressure. For some rubber compounds, an additional external pressure build-up supports a successful vulcanization process. Therefore, the suitability of a pressure transfer must also be possible.

### 2.3. Media Requirements in Combination with Rubber Compounds

After the vulcanization process, the MDS should be easily removable from the finished rubber shape. Furthermore, the MDS must not infiltrate, interact, or diffuse into the rubber material. In general, no physical or chemical reactions may be caused in the rubber. Furthermore, the essential parameters for vulcanization are temperature, time, and pressure. Embedding rubber in an MDS significantly influences the course of the heating time until the vulcanization temperature is reached. The influence differs depending on the MDS. The times for the start and the end of vulcanization have to be redetermined, as these have a direct effect on the mechanical properties.

## 3. Materials and Methods

### 3.1. Media Selection

The identification of appropriate media for the vulcanization process as well as feeding this medium into the 3D printing process is challenging. The current study is initially focused on the dimensional stabilization in the vulcanization process. Therefore, only media that can theoretically be integrated into a 3D printing process and that enclose the rubber shape well are selected. Typical thermoplastics like polylactide (PLA), acrylonitrile butadiene styrene (ABS), polyethylene terephthalate-glycol (PETG), or thermoplastic polyurethane (TPU) for FDM printing are processed in a temperature range from 180 to 250 °C [25]. However, these materials are at risk for melting or at least softening during the vulcanization process. In addition, difficulties are seen with regard to the removal from a thermoplastic mold. Therefore, these materials are not considered as suitable MDS. High-temperature thermoplastics such as polyether ether ketone (PEEK) (processing temperature above 340 °C [26]) and additively producible metals can lead to undesired and uneven vulcanization due to the high processing temperatures. These materials are also excluded for MDS. Primarily, media that are typically used for casting processes or impression material are considered.

The first medium is a 2-component liquid silicone rubber, which hardens after mixing. The selected silicone rubber is very heat resistant. In addition, due to the elastic behavior of both materials, a good separability is expected. The second medium is molding sand. This sand has rounded-edged grain shape, smooth grain surface as well as good pourability and is used for molds in aluminum and steel foundries. Based on these properties, the molding sand serves as a possible MDS. Plaster is the third medium. It is very accurate in shape and cures well. Depending on the type of plaster, curing times vary and can be adapted to the additive manufacturing process. Due to the high porosity of plaster, easy removal after vulcanization is expected. All media can enclose a rubber shape well in the state of processing (liquid or very fine-grained) and are sufficiently temperature stable. The selected media acting as MDS are listed in Table 1 with their advantages and possible disadvantages for the additive manufacturing process of rubber.

**Table 1:** Selected media serving as media for dimensional stabilization (MDS) with properties, advantages, and possible disadvantages for the additive manufacturing process of rubber.

Medium	Type	Usage in the 3D Printing Process	
		Positive Properties	Possible Disadvantages
Silicone rubber	TFC silicone rubber type 3 [27]	Good flowability High-temperature resistant (up to 450 °C)	Hardening time too long for dimensional stabilization Too soft for dimensional stability
Molding sand	Oil-bound molding sand [28]	Reusable No hardening time Good pourability (grain size 0–1 mm)	Discharge via an extruder still has to be conceptualized
Plaster	Plaster [29]	Good flowability High shape accuracy	Hardening period must be well coordinated in the manufacturing process

In previous studies, the synthetic rubber acrylonitrile butadiene rubber (NBR) has proven to have excellent printability [9] and is therefore used for the vulcanization tests. The rubber compound is commercially available and is procured by the company Kraiburg [30]. Properties of the rubber compound NBR are listed below:

- Mooney viscosity (ML1 + 4; 100 °C): 37
- Vulcanization Conditions Dumbbell specimen S2 (10 min): 170 °C
- Essential properties: high abrasion resistance, high tear resistance, high tensile strength, high resistance to compression deformation.

### 3.2. Experimental Tests

In the experimental tests, unvulcanized rubber components are embedded and vulcanized in the MDS. The aim of the vulcanization tests is examination of:

- Thermal behavior of the MDS and effect on the heating time inside the rubber samples,
- Removability of MDS from rubber after vulcanization,
- Influence on dimensional stabilization, and
- Effects on surfaces.

For this purpose, two different tests are conducted: vulcanization tests with an evaluation of heating curves inside the rubber specimens (see Section 3.2.1) and tests on the dimensional stabilization on additively manufactured rubber parts (see Section 3.2.2).

#### 3.2.1. Evaluation of Heating Curves

**Figure 2:**
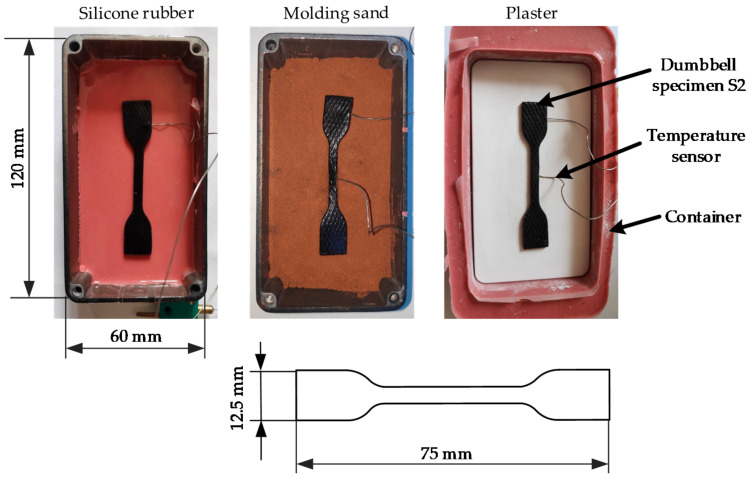
Dumbbell specimen with temperature sensors on 10 mm base layers of MDS.

In order to obtain fully vulcanized rubbers after the vulcanization process, the required vulcanization time within the media must first be determined. For this purpose, dumbbell specimens of type S2 are die-cut out of the rubber compound, as shown in Figure 2. The shape is selected due to follow-up investigations to analyze the potential effects of MDS on material properties. Tests on the properties of final rubbers, like tensile strength or tear resistance, are usually carried out with these dumbbell specimens [31]. For the vulcanization tests, the dumbbell specimens are embedded in the MDS. First, a 10 mm high base layer is prepared in a container. Afterwards, one temperature sensor (Thermocouple Type K [32]) is inserted in the middle and one at the end of the dumbbell specimen, as shown in Figure 2. Then, the samples are embedded with the MDS by the thickness of the specimen (2 mm) plus another 10 mm layer. Thus, the samples are surrounded with a 10 mm MDS layer on the top and bottom. The silicone rubber and the plaster are heated without the container. In contrast to that, the molding sand is heated with a metal container (2 mm wall thickness) but without cover on the top. In all cases, vulcanization was conducted at 170 °C in a preheated convection oven (Model BD 115 [33]) (Binder Gmbh, Tuttlingen, Germany) while recording the temperature curves. The focus of this test is the heat conduction of the MDS until the target vulcanization temperature is reached in the rubber sample.

#### 3.2.2. Dimensional Stabilization

**Figure 3:**
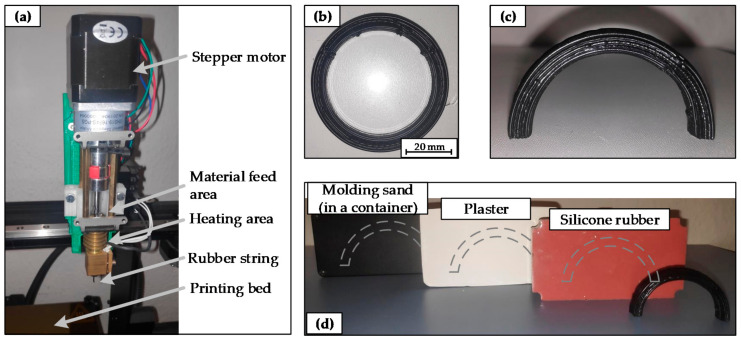
(**a**) Screw extruder for the additive manufacturing for rubber compounds; (**b**) additive manufactured rubber disc, (**c**) unvulcanized arc sample for the vulcanization tests, and (**d**) in MDS embedded arc samples for the vulcanization tests.

In order to demonstrate the necessity and proof of dimensional stabilization, the flat dumbbell specimen is not a suitable object. Therefore, an additively manufactured sample is used, which significantly deforms the shape during the vulcanization. The sample is produced with an FDM printer (Makergear ID3, Beachwood, OH, USA), where the conventional extruder has been replaced with a screw extruder, see also [17]. The setup is shown in Figure 3a. At present, there are only limited shapes that can be produced with the current technology. In particular, only 2D shapes are possible, as long as no support medium is used within the printing process. In addition, previous investigations have shown that strand deposits with changes in direction of sharp corners result in increased manufacturing deviations. For this reason, a circular rubber sample is selected for the additive manufacturing process. In order to be able to demonstrate the dimensional stability, a self-supporting structure is to be tested in the vulcanization process. Therefore, a circular disc (outer diameter 60 mm) with a hole (inner diameter 50 mm) and a height of 8 mm is manufactured by a layer-wise built up with circular paths in each layer. If the height is increased further, higher inaccuracies occur during strand laydown. The main parameters for the additive manufacturing process are listed below:

- Nozzle diameter: 0.8 mm
- Extrusion multiplier: 4 mm
- Layer height: 0.4 mm
- Infill: 100%
- Extrusion temperature: 70 °C
- Printing speed: 25 mm/s
- Printing time: 18 min (one piece).

Once the rubber disc is produced (Figure 3b), it is cut in half and set up with the cut surfaces facing downwards. In this way, an arc is finally obtained as a self-supporting structure, as shown in Figure 3c. Subsequently, the arc samples are embedded in the MDS in the same way as the dumbbell specimens with a 10 mm MDS layer on the top and bottom. The heating time identified in the first tests is used to cross-link these samples. Figure 3d shows the samples prepared for the vulcanization tests. One arc sample is vulcanized without MDS, which serves as a comparison in the evaluation of dimensional stabilization. In addition, all samples with MDS are analyzed regarding the removability of the MDS and its influence on the rubber surfaces.

## 4. Results and Discussion

### 4.1. Thermal Behavior of the Media and the Effects on the Vulcanization Time

**Figure 4:**
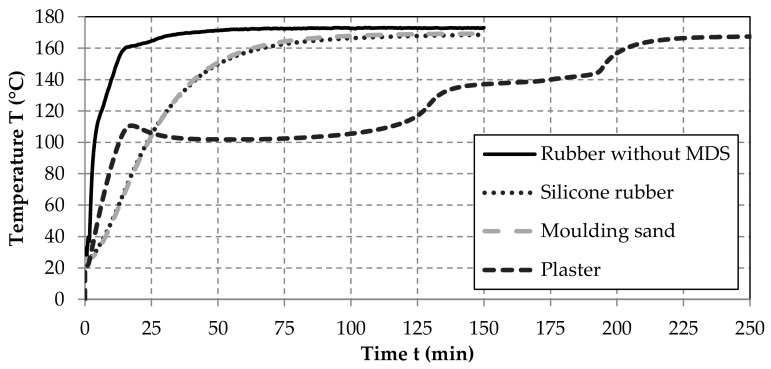
Time curves until the vulcanization temperature is reached inside the dumbbell specimen.

Figure 4 illustrates the temperature curves of the samples in the MDS during the vulcanization process. The curves for the measurement in the middle and on the outer side of the sample are very close to each other and partly on top of each other. Therefore, the results for the measured values are displayed only in the middle of the samples. As a comparison, a temperature curve of a dumbbell specimen without a medium was recorded as well. As expected, the heating time until reaching the vulcanization temperature is considerably extended by the MDS. The rubber sample without MDS reached the temperature of 160 °C after 15 min and the target value of 170 °C ± 1 K after about 35 min. The time curves for the silicone rubber and the molding sand are very similar and exceed 160 °C after about 60 min. The 170 °C are not reached in any of those two tests. Instead, a maximum temperature of 168 °C was observed. In order to achieve the desired degree of cross-linking, adjustments for the vulcanization process must be made accordingly. If further tests show that the selected media are suitable for the vulcanization process, further investigations are necessary regarding the relationship of the thickness of the MDS cover layers and cross-linking of the rubber. To this end, the specific vulcanization curves for the rubber materials must be determined. This can be done by different methods of thermal and mechanical analysis, like differential scanning calorimetry or different types of curemeters (see [34]). Moreover, further investigations are necessary regarding the impact of the thickness and the thermal properties of the MDS cover layers on the heating curves inside rubber samples of different shape. In this regard, numerical simulations, like, e.g., the applied in [35], can help to determine the necessary heating strategies required for the vulcanization of the embedded rubber samples.

The heating curves of rubber samples inside plaster shows a different behavior, which also occurred in a repeat test. Above a temperature of 100 °C, the crystal water contained in the plaster withdraws, which is also called burning. During this process, temperature plateaus are maintained, as shown in Figure 4. As a result, the rubber sample reaches the temperature of 160 °C only after 200 min. As for the other media, a maximum temperature of only 168 °C is finally reached. If the plaster should prove to be particularly suitable for the further MDS requirements, the vulcanization process should be designed very specifically or should be adjusted with a longer holding time for the cross-linking around 120 °C.

### 4.2. Vulcanization of Arc Rubber Shapes

**Figure 5:**
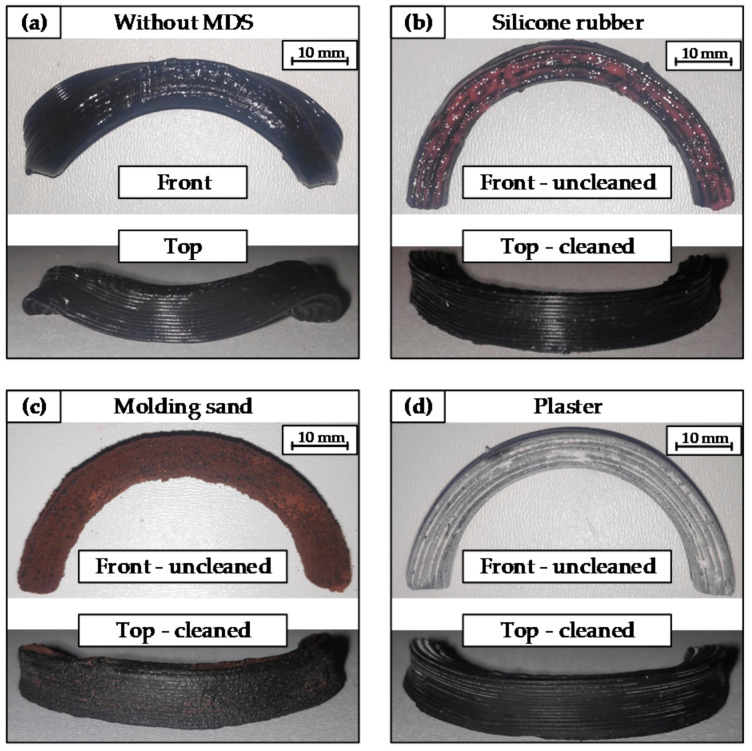
Vulcanized arc samples with the uncleaned front view and in the cleaned state with the top view: (**a**) without MDS, (**b**) silicone rubber, (**c**) molding sand, and (**d**) plaster.

Based on the findings on vulcanization (see Section 3.1), the embedded arc samples are vulcanized for 120 min (molding sand and silicone rubber) and 225 min (plaster). The results for the respective samples are shown in Figure 5.

#### 4.2.1. Results for the Removability of Media

Removing the samples from the MDS is easy for all media. The sand can simply be crumbled, the silicone is sliced open, and the plaster can be broken up. The photos in Figure 5 show the conditions right after removal from the media without further cleaning in the front view. The cleaned states of the samples are shown in Figure 5 in the top view.

In the case of molding sand and plaster, it is difficult to remove these residues between the layers. Additionally, NBR rubber seems to react with the selected silicone rubber, since a silicone layer directly adjacent to the NBR material has not hardened. Instead, it now forms a greasy film around the rubber, which is difficult to remove. This could also be observed with the dumbbell specimen in the silicone mold. As an additional test, an EPDM rubber was embedded in the silicone rubber. The silicone rubber is completely cured with this material. Therefore, this seems to be only a specific reaction between the selected silicone rubber and the NBR rubber. If silicone rubber is chosen as MDS, the reactivity between silicone rubber and the rubber system used should be considered.

#### 4.2.2. Results for Dimensional Stability

The arc sample without MDS (Figure 5a) collapsed during the vulcanization process. In contrast, it can be determined for all test samples with MDS that the dimensional stability of the arc has been maintained. The individual print layers are still clearly visible with the silicone rubber and plaster. However, for the molding sand, the visibility of the layers is no longer clearly given.

#### 4.2.3. Effects on the Surface

The results for the rubber surfaces turned out differently for each MDS. For the silicone rubber, the surface of the individual layers was smooth and glossy (Figure 5b), just like the sample without MDS. The sample with plaster also had a smooth surface, but after vulcanization and cleaning, it was not shiny but rather matte (Figure 5d). The situation was quite different with the sample in the molding sand. The surface that was glossy at the beginning showed a significant roughness of the vulcanized rubber surface after vulcanization process (Figure 5c). Although the molding sand is fine-grained, a completely smooth surface to the rubber could not be formed. Powdered and sandy materials will always have a corresponding roughness in the direction of the rubber surface. Nevertheless, future work should deal with more fine-grained sands to minimize the influence on surface roughness. In general, however, this effect can be used to selectively adjust the surface roughness on the final rubber component by varying the particle size, e.g., to achieve special friction values with other surfaces in the application.

### 4.3. Suitability of the Selected Media as MDS

All selected media showed different advantages and disadvantages for a possible use as MDS in an additive manufacturing process for rubbers. Table 2 summarizes the observed effects of the vulcanization tests. In general, none of the media can be excluded, but also none can be used directly as MDS. All media were able to maintain the main shape of the arc very well during the vulcanization process. With silicone rubber, it was found that there is a risk of reaction with the rubber compounds. Complete curing around the rubber shape was prevented for the silicone rubber (Figure 5b). This resulted in a more extensive cleaning process. Influences on the mechanical rubber properties are also very likely due to the reaction. The reaction with other rubber compounds and other silicone rubbers should be investigated in further studies.

Due to the reaction during heating, the plaster makes the design of the vulcanization process considerably more challenging (Figure 4). Furthermore, it was difficult to remove the plaster residues from the cavities of vulcanized rubber. Another effect was seen in the non-typical matte surface of the vulcanized samples (Figure 5d).

The molding sand mainly influences the surface quality (Figure 5c). In addition, the removal from the cavities of the vulcanized rubbers involves a lot of effort. However, this fact can be used specifically to create preferred surface finishes.

## 5. Conclusions

The paper investigated the suitability of media to stabilize rubber forms during the additive manufacturing and vulcanization process. After describing the concept of additive manufacturing of rubbers via extrusion-based 3D printing, the first essential requirements for MDS were derived. Based on the concept, the different media types silicone rubber, molding sand, and plaster were selected as possible media for dimensional stabilization (MDS) and were examined in experimental vulcanization tests. All media types could maintain the general shape of selected rubber samples. However, each medium showed different effects on the NBR rubber compound in terms of the vulcanization process, interaction between the media, removal after vulcanization, and on surface characteristics. In summary, none of the media can be described as unsuitable, because the different effects of the media can also be used specifically to set certain properties for the final rubber product.

Further research should investigate the feeding of the media in the additive manufacturing process on the one hand and examine the influences on the rubber properties on the other hand. In addition, the test setup must be optimized in order to be able to examine further rubber compounds in combination with the MDS in the additive manufacturing and vulcanization process. Furthermore, due to the high influence on the heating time, further tests are required to set targeted degrees of cross-linking as well as to test the suitability of pressure transfer necessary for some rubber compounds. Numerical simulations can be developed with the obtained data and used to simulate the vulcanization behavior of rubber in MDS. This provides the basis for uniformly vulcanized samples and for performing tests to compare mechanical properties.

## Figures and Tables

**Table 2:** Evaluation matrix for the suitability of the selected media as MDS.

	Silicone Rubber	Molding Sand	Plaster
Vulcanization process	Continuous heating	Continuous heating	Heating with plateaus
Extraction from MDS	Without issues	Without issues	Without issues
Removal of residues	Simple	Difficult	Very difficult
Dimensional stability (of the arc shape)	Well preserved	Well preserved (without the surface)	Well preserved
Surfaces	Glossy/smooth	Matt/rough	Slightly glossy/smooth
Other Effects	No curing around the rubber	3D printing layers no longer recognizable	None
